# Intelligence

**Published:** 1832-02

**Authors:** 


### INTELLIGENCE.

### MEDICO-CHIRURGICAL SOCIETY, JANUARY 24.

*Account of some Experiments on the use of Styptics in Hemorrhage;* by Cæsar Hawkins, Esq.

This paper gave an account of some experiments made by Mr. Hawkins, at St. George's Hospital, in order to shew the pupils of that establishment, (who had witnessed some experiments of MM. Talrich and Halma Grand with their new styptic,) the real principles to which Mr. Hawkins thought the cessation of the hemorrhage ought to be ascribed. In some experiments, Mr. Hawkins used small compresses in the manner that the new styptic is used by MM. T. and H., but dipped in other liquids, decoction of galls, Ruspini's styptic, and plain water. Some other experiments were made like those of Dr. Jones, by merely closing the skin and allowing the blood to form a diffused aneurism. The result was, that, in some of the experiments, the hemorrhage was stopped permanently; in two others, secondary hemorrhage took place on the second and third days, and the animals died from repeated loss of blood, no sufficient coagulum having formed in the arteries. In one of the successful experiments, the artery was pervious, the wound having completely healed; in the others, the arteries were filled with coagula; and preparations of the vessels were shewn to the society.

The conclusions to which Mr. Hawkins came were, that simple pressure for ten minutes in an *open wound* would check hemorrhage as effectually as Dr. Jones had shewn it could be stopped when the wound in the skin was *closed*, without any direct pressure on the artery; that the hemorrhage was stopped by the same means as in Dr. Jones's experiment, by the formation of coagula and the effusion of lymph; that *natural means alone* would stop the bleeding from an artery, out of which a piece had been cut, as completely as Dr. Jones had shewn it could be done, when punctures or longitudinal or transverse wounds had been inflicted; that, when styptics were employed, the formation of coagula was principally owing to the *pressure*, and in a minor degree only to the *styptic;* since, when Ruspini's styptic and water were used, which do *not* coagulate blood, the hemorrhage stopped, as well as when galls or the new styptics, which *do* coagulate blood, were employed; and that, as Dr. Jones's and M. Halma Grand's, as well as Mr. Hawkins's own experiments, shew that the result is uncertain, even in brutes, when no ligature is used in partial wounds of large arteries, but that secondary hemorrhage is liable to occur at various periods, *a fortiori*, no reliance should be placed either on styptics or on pressure, if circumstances allow of the ligature being used in cases where a large artery is wounded in man.

---

*Styptic Powder*. At a recent sitting of the Académie de Médecine, M. Bonafous read a paper descriptive of a new powder, which is said to be capable of atresting hemorrhage from the largest arterial trunks in consequence of accidental or surgical wounds; this powder is composed of equal parts of resin, charcoal, and gum arabic. M. B. related several cases, from which it appears that this composition, when applied to the divided brachial artery of a man, and to the smaller arteries of the arm, to leech bites, to the wounded carotid and tibial artery of a horse, &c., completely arrested the bleeding. The powder is applied upon a small roll of lint, which is removed at the expiration of two or three days, when complete occlusion of the wounded vessel will be found to have taken place.—*Bull. des. Sc. Med.*

Whether the precise composition recommended by M. Bonafous has ever before been employed as a styptic, we know not; but we may remark, that powdered resin is highly eulogised by many old surgical writers for its power of stopping severe hemorrhages from wounded arteries, &c ; but that it has long been laid aside, from a conviction of its inefficacy.—Editor.

---

### LITERARY INTELLIGENCE.

Preparing for publication, A Clinical Report of the Royal Dispensary for Diseases of the Ear, with Observations on the Deaf and Dumb, by J. H. Curtis, Esq.

In the press, and speedily will be published, A Dictionary of Practical Medicine; comprising General Pathology, the Nature and Treatment of Diseases, Morbid Structures, and the Disorders especially incidental to Climates, to the Sex, and to the different Epochs of Life; with numerous approved Formulæ of the Medicines recommended; by J. Copland, M.D. (Uniform with Cooper's Surgical Dictionary.)

### METEOROLOGICAL JOURNAL,

**By Messrs HARRIS and Co. Mathematical Instrument Makers, 50, High Holborn.**

| | Moon. | Rain gauge. | Thermometer. | | | Barometer. | | De Luc's Hygrometer | | Winds. | | Atmospheric Variation. | | |
|---|---|---|---|---|---|---|---|---|---|---|---|---|---|---|
| Dec. | | | 9 a.m. | max. | min. | 9 a.m. | 10 p.m. | 10 p.m. | 9 a.m. | 9 a.m. | 10 p.m. | 9 a.m. | 2 p.m. | 10 p.m. |
| 20 | | | 44 | 44 | 42 | 29.59 | 29.48 | 65 | 67 | SSW | WSW | foggy | cloudy | rain |
| 21 | | | 43 | 45 | 38 | .49 | .64 | 67 | 69 | W | WSW | | | fine |
| 22 | | | 43 | 47 | 38 | .64 | .45 | 68 | 66 | SSW | WSW | | foggy | |
| 23 | | | 43 | 44 | 35 | .66 | .90 | 66 | 67 | WNW | NW | | | |
| 24 | | | 37 | 40 | 32 | 30.05 | 30.14 | 67 | 68 | WNW | WNW | | cloudy | |
| 25 | ☾ | | 35 | 38 | 30 | .14 | .14 | 68 | 68 | NW | NW | fog | fog | fog |
| 26 | | | 35 | 39 | 35 | .11 | .14 | 68 | 70 | SSW | NNW | foggy | foggy | |
| 27 | | | 38 | 40 | 34 | .23 | .25 | 70 | 68 | NNW | N | | | cloudy |
| 28 | | | 37 | 40 | 36 | .23 | .23 | 70 | 71 | NNE | N | | cloudy | rain |
| 29 | | | 40 | 39 | 36 | .11 | .04 | 71 | 69 | NE | NNE | | | cloudy |
| 30 | | | 36 | 37 | 34 | .04 | 30.00 | 69 | 69 | E | NNE | | fine | fine |
| 31 | | | 37 | 39 | 31 | .06 | .06 | 66 | 65 | NE | E | fine | | |
| Jan. 1 | | | 33 | 34 | 30 | 29. | 29. | 65 | 66 | NE | NE | | | |
| 2 | | | 34 | 35 | 32 | .78 | .66 | 67 | 66 | ESE | NE | foggy | | |
| 3 | ● | | 32 | 34 | 29 | .64 | .66 | 66 | 67 | WSW | WSW | | cloudy | foggy |
| 4 | | | 32 | 35 | 32 | .54 | .51 | 67 | 66 | SSW | S | fog | foggy | fine |
| 5 | | | 33 | 33 | 30 | .49 | .45 | 66 | 68 | E | SE | foggy | | foggy |
| 6 | | | 37 | 40 | 37 | .34 | .31 | 69 | 69 | ESE | ESE | | fine | cloudy |
| 7 | | | 39 | 41 | 35 | .18 | .20 | 71 | 71 | SSE | ESE | | cloudy | rain |
| 8 | | | 38 | 41 | 37 | .24 | .31 | 73 | 72 | E | E | fine | cloudy | foggy |
| 9 | | | 41 | 45 | 39 | .33 | .22 | 72 | 73 | SE | ESE | fog | rain | rain |
| 10 | | | 43 | 47 | 40 | .47 | .54 | 72 | 72 | W | SW | foggy | rain | cloudy |
| 11 | ☽ | | 47 | 49 | 42 | .60 | .48 | 70 | 70 | SW | W | cloudy | | fine |
| 12 | | | 45 | 46 | 40 | .64 | .44 | 68 | 69 | W | SW | foggy | cloudy | rain |
| 13 | | | 42 | 42 | 33 | .40 | .57 | 69 | 67 | WNW | NE | | | fine |
| 14 | | | 36 | 38 | 32 | .96 | 30.11 | 65 | 67 | N | NNE | fine | fine | |
| 15 | | | 36 | 40 | 30 | 29.19 | .24 | 67 | 65 | NNE | NNE | | foggy | |
| 16 | | | 34 | 37 | 35 | .27 | .22 | 65 | 66 | SW | W | foggy | | foggy |
| 17 | ○ | | 37 | 42 | 34 | .15 | .14 | 67 | 67 | WSW | W | | cloudy | |
| 18 | | | 38 | 41 | 36 | .17 | .17 | 67 | 68 | W | W | | fine | |
| 19 | | | 37 | 37 | 31 | .17 | .07 | 68 | 68 | SW | SW | fog | foggy | |

The Rain gauge having been frozen, the quantity cannot be given.

